# Recombinant Modified Vaccinia Virus Ankara Expressing Glycoprotein E2 of Chikungunya Virus Protects AG129 Mice against Lethal Challenge

**DOI:** 10.1371/journal.pntd.0003101

**Published:** 2014-09-04

**Authors:** Petra van den Doel, Asisa Volz, Jouke M. Roose, Varsha D. Sewbalaksing, Gorben P. Pijlman, Ingeborg van Middelkoop, Vincent Duiverman, Eva van de Wetering, Gerd Sutter, Albert D. M. E. Osterhaus, Byron E. E. Martina

**Affiliations:** 1 Department of Viroscience, Erasmus Medical Center, Rotterdam, The Netherlands; 2 Institute for Infectious Diseases and Zoonoses, University of Munich LMU, Munich, Germany; 3 Laboratory of Virology, Wageningen University, Wageningen, The Netherlands; 4 Erasmus Medical Center Laboratory Animal Science Center (EDC), Rotterdam, The Netherlands; 5 Artemis One Health, Utrecht, The Netherlands; Centers for Disease Control and Prevention, United States of America

## Abstract

Chikungunya virus (CHIKV) infection is characterized by rash, acute high fever, chills, headache, nausea, photophobia, vomiting, and severe polyarthralgia. There is evidence that arthralgia can persist for years and result in long-term discomfort. Neurologic disease with fatal outcome has been documented, although at low incidences. The CHIKV RNA genome encodes five structural proteins (C, E1, E2, E3 and 6K). The E1 spike protein drives the fusion process within the cytoplasm, while the E2 protein is believed to interact with cellular receptors and therefore most probably constitutes the target of neutralizing antibodies. We have constructed recombinant Modified Vaccinia Ankara (MVA) expressing E3E2, 6KE1, or the entire CHIKV envelope polyprotein cassette E3E26KE1. MVA is an appropriate platform because of its demonstrated clinical safety and its suitability for expression of various heterologous proteins. After completing the immunization scheme, animals were challenged with CHIV-S27. Immunization of AG129 mice with MVAs expressing E2 or E3E26KE1 elicited neutralizing antibodies in all animals and provided 100% protection against lethal disease. In contrast, 75% of the animals immunized with 6KE1 were protected against lethal infection. In conclusion, MVA expressing the glycoprotein E2 of CHIKV represents as an immunogenic and effective candidate vaccine against CHIKV infections.

## Author Summary

The Chikungunya virus (CHIKV) represents a threat to Europe. This is supported by its recent introduction and autochthonous transmission in Italy and France in 2007 and 2010 respectively. CHIKV causes an acute febrile illness and severe joint pains that may last for several months in some patients. In absence of a proven effective treatment, safe and effective vaccines represent the best way to control outbreaks of CHIKV. This study describes the development of a vaccine candidate based on the Modified Vaccinia Ankara virus (MVA). MVA has been used to develop vaccines against several viruses and based on the safety and efficacy records of MVA vaccines, various candidate vaccines are currently in clinical trials. MVA expressing structural proteins of CHIKV were constructed and tested in AG129 mice for immunogenicity and protective ability. All animals that received MVA expressing E2 developed neutralizing antibodies and were protected against lethal challenge. Our study shows that recombinant MVA expressing the E2 protein of CHIKV represents a potential candidate vaccine for prevention of CHIKV infections in travellers or endemic areas.

## Introduction

Chikungunya virus (CHIKV) belongs to the family *Togaviridae*, genus Alphavirus. The virus was first isolated in 1952 during an epidemic of arthralgic disease in Tanzania, and CHIKV is now known as a mosquito-borne virus endemic in many parts of Sub-Saharan Africa as well as in Asia [1]. Epidemic CHIKV is maintained mainly as an urban transmission cycle, involving humans as amplification hosts and the peri-domestic mosquitoes *Aedes aegypti*, and since recently also *Aedes albopictus*. Large outbreaks have been reported since 2004, with the first description originating from Kenya [2], [3] and subsequent introduction of the virus to islands in the Indian Ocean [4], [5] and India [6], [7]. These epidemics resulted in up to six million cases, with regular importation of the virus in Europe, Southeast Asia and the Americas [8]. The first autochthonous CHIKV infections in Europe occurred in Italy in 2007 and France in 2010. The widespread dissemination of CHIKV is associated with amino acid mutations in the glycoprotein E1 (A226V), which facilitated adaptation of CHIKV to the *Aedes albopictus*, a mosquito that survives in temperate climates and is widely distributed [9].

While patients with CHIKV infection may exhibit a variety of symptoms such as rash, chills, headache, nausea, photophobia, and vomiting, it is characterized by acute high fever, and severe polyarthralgia [10]. Persistent arthralgia can remain for years [11]–[14] and it has been shown to be a major cause of long-term discomfort [15]. Fatalities following CHIKV infection are rare, but have been shown to occur [16]–[18] as a result of severe neurologic disease [19]–[21]. To date, CHIKV continues to pose a threat to several countries in the world and continues to cause outbreaks in India and Southeast Asia, resulting in persistent morbidity and consequently considerable economic losses. Therefore, vaccine development remains a high priority. Currently, there are no licensed CHIKV vaccines available, although there are several experimental candidate vaccines are under investigation.

CHIKV is composed of a single-stranded, positive-sense RNA genome of about 12 kb and contains two open-reading frames (ORFs). These two ORFs encode four non-structural proteins (nsP1, nsP2, nsP3 and nsP4), which are important for virus replication, and five structural proteins (C, E1, E2, E3 and 6K). The capsid (C) proteins surround the RNA molecule and together form the nucleocapsid. The E1/E2 heterodimers are embedded in the viral membrane and the average CHIKV particle contains around 80 E1/E2 spikes projecting from the viral envelope. Alphaviruses enter susceptible cells after attachment to cellular receptors on many different cell types. The E1 spike protein drives the fusion process within the cytoplasm, while the E2 protein is believed to interact with cellular receptors and therefore most probably constitute the target of neutralizing antibodies [22]–[24].

Recombinant Modified Vaccinia virus Ankara (MVA) is among the most promising live viral vector systems, because of its well-established biological and clinical safety, together with its suitability for expression of various heterologous proteins. MVA was originally developed as a safe and effective vaccine against smallpox by employing serial passages in primary chicken embryo cells [25], [26]. After more than 570 passages in avian tissue culture MVA had lost the ability to undergo multiple rounds of productive replication in a broad range animal species including humans [27]. Furthermore, when used as viral vaccine MVA has been shown to induce immune responses against many different recombinant antigens and provide protection against multiple viral diseases [28]. The immunogenicity of recombinant proteins is higher or at least similar to what is achieved during a productive infection with replicating vaccinia virus vectors [29] and makes MVA an attractive platform for vaccine development.

Here we describe for the first time the construction and evaluation of three different recombinant modified vaccinia Ankara (MVA) viruses expressing 6KE1, E3E2, or the entire CHIKV envelope polyprotein E3E26KE1. The three vaccine candidates were evaluated in the AG129 mouse model to assess induction of protective immunity against CHIKV-S27.

## Materials and Methods

### Ethics statement

The animal experiments described in this paper were conducted according to Dutch guidelines for animal experimentation after approval (EMC 2514, 122-11-27) by the Animal Welfare Committee of the Erasmus Medical Centre, Rotterdam, The Netherlands. Research plans were reviewed by the local ethical review committee, which considered the benefit of an experiment against the cost of distress inflicted to the animals during the experiments. Appropriate practices and procedures as defined in the Biosafety in microbiological and biomedical laboratories (US Dept. of Health and Human Services) were used in sample handling. Samples were stored at −80°C in a biosafety level-3 (BSL-3) facility at the Erasmus Medical Centre Rotterdam, The Netherlands.

### Cells and viruses

Vero E6 cells were cultured in DMEM with 10% heat inactivated fetal bovine serum (HI-FBS), supplemented with penicillin/streptomycin/L-glutamine (2 mM) (psg; Biowhittaker for all), sodium bicarbonate and 10 mM hepes buffer. Baby hamster kidney BHK-21 cells (clone 13, ATCC CCL-10) were grown in DMEM supplemented with 10% HI-FBS and psg. *Aedes pseudoscutelaris* insect cells (AP-61) were grown in Leibovitz-15 medium (BioWhittaker) supplemented with 5% tryptose phosphate broth medium (MP Biomedicals), psg and 5% HI-FBS. Cells were grown and maintained at 27°C. Primary chicken embryo fibroblast cells (CEF) were isolated from 11-day old embryonated chicken eggs and maintained in DMEM (BioWhittaker) supplemented with psg, non-essential amino acids (Sigma) and 10% HI-FBS.

The African prototype CHIKV-S27 strain (genbank acc.nr. AF369024), the CHIKV-IND/NL10, and LS3 [30] strains were grown on Vero E6 cells and virus titers were determined after titration of virus stocks on Vero E6 cells. To this end, 96-wells plates (greiner) were seeded with 5×10^4^ Vero cells per well. A serial ten-fold dilution of virus stocks was made in octaplicates on the seeded plates, and plates were subsequently incubated for four days at 37°C. The Vero E6 plates were scored microscopically for cytopathic changes (cpe). Viral titers were expressed as TCID_50_ per ml as calculated with the Kärber method [31]. The highly attenuated vaccinia virus (VACV) strain MVA (clonal isolate F6, CEF passage 584) was used for this study. MVA is derived from VACV chorioallantois virus Ankara (CVA) by more than 570 passages in CEF. During this serial passage MVA incurred six major deletions and many smaller mutations in its genome and developed a severe restriction in host range so that it is unable to replicate in most mammalian cells [32], [33].

### Generation of recombinant virus

RNA of the African prototype CHIKV-S27 strain was isolated using the High Pure RNA isolation kit (Roche). cDNA of 6KE1, E3E2, and E3E26KE1 were obtained with SuperscriptIII (Invitrogen) and the respective products were amplified with high fidelity polymerase PFU (Roche). Primers were flanked by BamHI and NotI restriction sites (Italic and underlined nucleotides). The following primers were used for specific amplification of 6KE1 (Fw: CGC*GGATCC*GCCGCCACCATGGCGGCCACATACCAAGAG; Rev: AGCTTT*GTTTAAAC*TTAGTGCCTGCTGAACGACACGCA), E3E2 (Fw: CGC*GGATCC*GCCGCCACCATGGAAGAGTGGAGTCTTG; Rev: AGCTTT*GTTTAAAC*TTATTTAGCTGTTCTGATGCAGCA), and E3E26KE1 (Fw: CGC*GGATCC*GCCGCCACCATGGAAGAGTGGAGTCTTG; Rev: AGCTTTGTTTAAACTTAGTGCCTGCTGAACGACACGCA). The resulting PCR products were cloned directionally into the MVA vector plasmid pIIIRedH5 and placed under the transcriptional control of the modified vaccinia virus early/late promoter PmH5 [34].

Recombinant MVA expressing 6KE1, E3E2, and E3E26KE1 (MVA-6KE1, MVA-E3E2, MVA-E3E26KE1) were generated using the principles of homologous recombination targeting the site of major deletion III within the MVA genome for insertion of recombinant gene sequences. Primary CEF were infected with MVA followed by transfection of the infected cells with pIIIRedH5 DNA, using FuGENE transfection reagent (Roche). Clonal isolates of recombinant MVA were obtained by multiple rounds of plaques purification on CEF cells and screening for the transient expression of the fluorescent marker mCHERRY. In each round, the MVA genome at the insertion site was analyzed by PCR and recombinant gene expression was confirmed by immunostaining of BHK-21 cells infected with selected recombinant viruses. Clonal recombinant MVAs expressing mCHERRY were further propagated on CEF until loss of the transient marker mCHERRY. To this end, limiting dilutions of the recombinant viruses were done and wells with the highest virus dilution displaying cpe were selected for further analysis. To generate the vaccine batches for the pre-clinical animal studies, all recombinant MVA were propagated in a multistep amplification process on CEF cells, purified by ultracentrifugation through 20% sucrose, and reconstituted in TE buffer, pH 9.0. Expression of the respective genes was confirmed by Western blot analysis of lysates of infected BHK-21 cells. Six different PCR primer sets were used to confirm the genetic integrity of the recombinant viruses (see Table S1). The sequence of all inserted recombinant genes was confirmed to be identical to the original CHIKV-S27 sequence using the 3130×l automatic sequencer (ABI).

### Immunoperoxidase staining

Cells were fixed in methanol, washed with PBS, blocked for endogenous peroxidase with hydrogen peroxide and after two washes with PBS cells were subsequently stained with polyclonal rabbit anti-E1 or rabbit anti-E2 [35] for 1 hour at 37°C. Cells were washed twice with PBS and primary antibody was detected using peroxidase labeled goat anti-rabbit IgG (Invitrogen) after incubation for 45 min at 37°C and 3-amino-9-ethylcarbazole (AEC) substrate for 10 min at RT. Amount of cells expressing the recombinant proteins were counted using a light microscope.

### Electron microscopy

To investigate whether the recombinant MVA-E3E26KE1 lead to generation of VLPs, BHK-21 and Hela cells were infected with the recombinant MVA, using an MOI of 1 and 10. Supernatant were collected 24 hours and 48 hours after infection. Supernatant were centrifuged for 10 minutes at 1200 rpm and 10 µl was used for EM analysis. Briefly, Copper 400 square mesh grids (Veco) were treated by Argon gas discharge and loaded with 5 µl culture fluid of MVA-infected cells for 30 sec at room temp. Excess liquid was removed using filter paper and the grids were treated with 2% uranyl acetate for 30 sec, excess uranyl acetate was carefully removed using filter paper. The grids were air dried and analyzed with a JEOL JEM 1011 transmission electron microscope at the Wageningen Electron Microscopy Centre.

### Vaccination—challenge experiments

Mice lacking the IFN-α/β/γ receptor (A129 mice) [36] have been described previously to be susceptible to CHIKV infection [37]. The AG129 mice were purchased from B&K Universal (UK) at the age of six weeks. Mice were maintained under specific pathogen-free conditions and were allowed to adjust to the facility for one week before experiments were performed. Four groups of mice (n = 8 for each group) were immunized with the MVA-6KE1, MVA-E3E2 and E3E26KE1 twice at 3-week intervals. Wildtype (wt) MVA was used as a negative control. All MVA stocks had a concentration of 10^8^ TCID_50_/ml and 50 µl was injected into the quadriceps muscles of the left leg of each animal. Blood was collected on day 0, 21, 63, 70 and 77.

Six weeks after the last immunization (day 63), all animals were challenged by intraperitoneal inoculation of 10^3^ TCID_50_ of CHIKV-S27 in a total volume of 100 µl and animals were checked daily for clinical signs of infection, such as lethargy and hind limb weakness. Animals were then sacrificed either 14 days post challenge or earlier if a humane end point (immobility and paralysis) was reached. At the end of the experiment, the survival rates were analyzed and compared between groups.

### Immunogenicity studies

To characterize the antibody responses triggered by immunization with the recombinant MVA vaccines, a virus neutralization test was performed. To this end, sera of immunized mice were heat-inactivated and diluted (1∶10 to 1∶2560) in triplicate in 96-wells plate and 100 TCID_50_ of CHIKV-S27, CHIKV-IND/NL10 or CHIKV-LS3 was added to each well. After one hour of incubation at 37°C, 1×10^4^ Vero E6 cells were added to each well and plates were incubated for another four days. Neutralizing titres were determined microscopically and expressed as the reciprocal of the highest serum dilution still giving 100% suppression of cpe.

### Determination of viral load

In order to quantify relative numbers of viral RNA in the respective tissues, 100 µL of organ homogenate was added to 400 µL of lysis buffer (Roche). Viral RNA was then extracted from spleen, liver, kidney, spinal cord, and brain samples using the automated MagnaPure method (Total nucleic acid isolation kit, Roche Diagnostics, the Netherlands) according to the manufacturer's instructions, and quantified using a one-step RT-PCR TaqMan protocol (EZ-kit, Applied Biosystems) and an ABI PRISM 7500 detection instrument. The primers and probe used for CHIKV RNA quantification were essentially as described [38]. Specifically, CHIKV-forw AAGCTCCGCGTCCTTTACCAAG; CHIKV-rev CCAAATTGTCCTGGTCTTCCT; and Probe: Fam-CCAATGTCTTCAGCCTGGACACCTTT-Tamra were used. Results are expressed as TCID_50_ equivalents per gram of tissue.

### Immuno-histochemistry

Tissues were removed and fixed in 10% neutral-buffered formalin, embedded in paraffin and sectioned at 4 µm. Slides were stained with hematoxylin and eosin (HE) and analyze by light microscopy. Subsequently, 4-µm thick paraffin sections were processed for immunohistochemistry. To this end, sections were deparaffinized in xylene, rehydrated in descending concentrations of ethanol and incubated for 10 min in 3% H_2_O_2_ diluted in PBS to block endogenous peroxidase activity. Antigen exposure was performed by incubation for 15 min at 121°C in citrate buffer (0.01 M, pH 6.0). Sections were incubated overnight at 4°C with rabbit-anti-CHIKV capsid (1∶5000), and the primary antibody was detected with secondary goat anti-rabbit IgG-PO (1∶100; Dako, The Netherlands). Sections were counterstained with Mayer's hematoxylin and mounted with Kaiser's glycerin-gelatin and analyzed using a light microscope.

### Statistical analysis

Differences in Kaplan-Meier survival curves between the groups were assessed using the log-rank test. All statistical analyses were performed using GraphPad Prism version 4 software (Graphpad Software, San Diego, USA). Differences between viral loads were assessed using the student's t test. P values≤0.05 were considered to be statistically significant.

## Results

### Characterization of the different candidate vaccines

**Figure 1:**
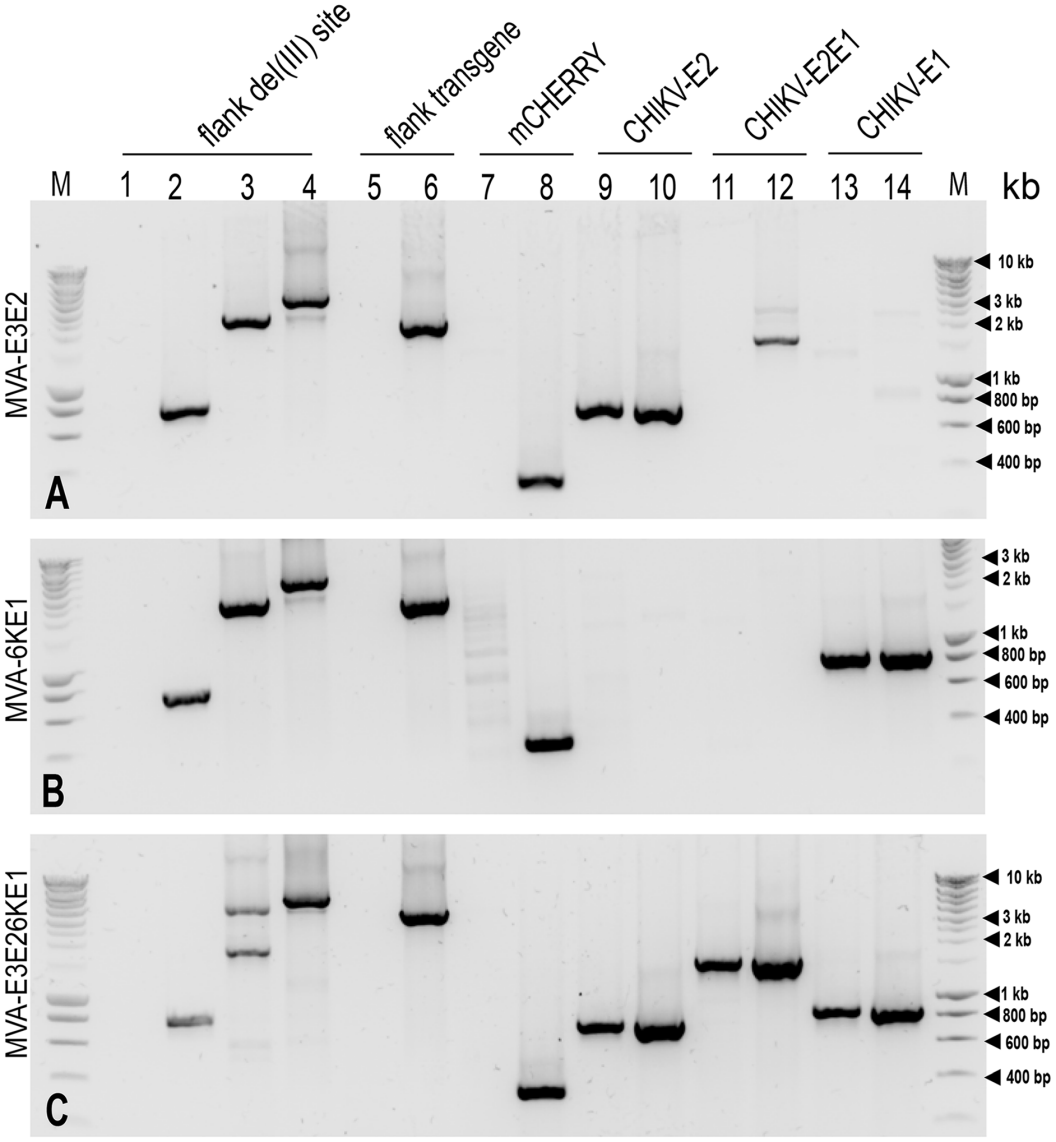
Analysis of candidate vaccines. Different MVA constructs were checked for presence of the specific transgene E3E2 (panel A), E3E26KE1 (panel B), and 6KE1 (panel C) using different sets of primers (Table S1). PCRs were conducted on DNA extracted from uninfected BHK-21 cells (lane 1, negative pcr), BHK-21 cells infected with wildtype MVA (lane 2) and recombinant MVA DNA (lanes 3, 5, 7, 9, 11 and 13), as positive controls shuttle vector containing the three respective transgenes (lane 4, 6, 8, 10,12 and 14) were used as a template. Amplicons were separated on 1% agarose gel with Sybrsafe staining and were visualised under blue light transillumination. M Smartladder, kb molecular weight.

**Figure 2:**
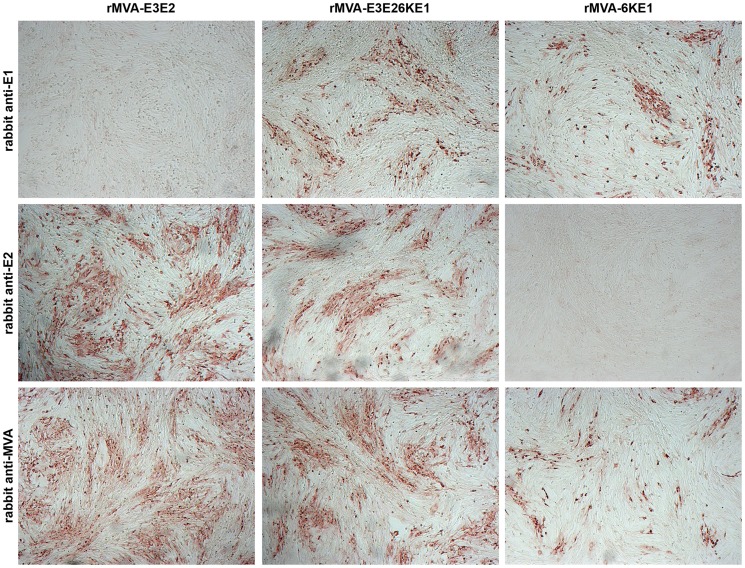
Detection of antigen expression. BHK-21 cells were infected with the respective candidate MVA vaccines and 24 hours later cells were fixed in methanol/acetone (1∶1) and stained for specific expression of CHIKV E1 (upper panel), E2 (middle panel) or MVA antigens (lower panel). The respective antigens were detected using rabbit polyclonal serum specific against E1, E2, and MVA as indicated. The staining confirmed the specific expression of the respective antigens and their purity. *Images were contrast enhanced in Adobe Photoshop.

The first step in generation of the candidate vaccines was selection of recombinant MVA that contained CHIKV-S27 gene sequences of interest. These recombinant viruses expressed the mCHERRY marker and contained the expected CHIKV gene. Next the recombinant viruses were passaged until they were mCHERRY-free and all constructs underwent thorough quality control PCR (Figure 1; Table S1). In previous studies, genetic instabilities have been recognized with MVA vector viruses that contain recombinant sequences encoding for heterologous viral glycoproteins [39], [40]. The genetic stability of final MVA-6KE1, MVA-E3E2 and E3E26KE1 recombinant viruses was evaluated after passing the viruses multiple times on CEF (data not shown) (Figure 1). Synthesis of the recombinant proteins E1 and E2 was confirmed in an immunostaining assay (Figure 2).

In order to investigate whether the MVA-E3E26KE1 construct resulted in production of VLPs, supernatant of cells infected with the MVA construct were collected several time points after infection. Extensive screening with electron microscopy revealed no presence of VLPs, suggesting that MVA-E3E26KE1 did not result in generation of VLPs.

### MVA-E3E2 and MVA-E3E26KE1 induce neutralizing antibodies against homologous and heterologous CHIKV

As shown in table 1, immunization with MVA-6KE1 and MVA-E3E2 induced low levels of neutralizing antibodies (range: 10–20) against both CHIKV-S27 and CHIKV-IND/NL10, 56 days post immunization. In contrast, recombinant MVA expressing the structural envelope cassette E3E26KE1 induced significantly higher levels of neutralizing antibodies compared to the other two candidate vaccines (P<0.05), with titers ranging from 40–160. Only MVA-E3E26KE1 induced neutralizing antibodies in 100% of the animals after one vaccination (day 21). Similar titers were obtained in neutralization assay using the LS2 strain (not shown).

**Table 1:** Neutralization titers against CHIKV-S27 and CHIKV-IND/NL10.

Animal #	Day 0	Day 21	Day 63	Day 0	Day 21	Day 63	Status after challenge
***MVA Vaccine***	***Challenge virus***						
**6KE1**	CHIKV-S27			CHIKV-IND/NL10			
Mouse 1	<10	<10	10	<10	<10	20	survived
Mouse 2	<10	<10	20	<10	<10	20	survived
Mouse 3	<10	<10	10	<10	<10	10	survived
Mouse 4	<10	<10	10	<10	<10	10	survived
Mouse 5	<10	<10	<10	<10	<10	<10	dead
Mouse 6	<10	<10	<10	<10	<10	10	survived
Mouse 7	<10	<10	<10	<10	<10	<10	survived
Mouse 8	<10	<10	10	<10	<10	10	dead
***MVA Vaccine***	***Challenge virus***						
**E3E2**	CHIKV-S27			CHIKV-IND/NL10			
Mouse 1	<10	10	20	<10	10	20	survived
Mouse 2	<10	<10	20	<10	10	20	survived
Mouse 3	<10	<10	20	<10	10	10	survived
Mouse 4	<10	<10	20	<10	10	20	survived
Mouse 5	<10	10	10	<10	10	20	survived
Mouse 6	<10	10	10	<10	10	10	survived
Mouse 7	<10	<10	10	<10	<10	10	survived
Mouse 8	<10	10	20	<10	10	20	survived
***MVA Vaccine***	***Challenge virus***						
**6KE1E3E2**	CHIKV-S27			CHIKV-IND/NL10			
Mouse 1	<10	10	80	<10	20	80	survived
Mouse 2	<10	10	80	<10	10	80	survived
Mouse 3	<10	10	160	<10	20	160	survived
Mouse 4	<10	10	80	<10	10	80	survived
Mouse 5	<10	20	40	<10	20	80	survived
Mouse 6	<10	10	40	<10	20	40	survived
Mouse 7	<10	20	80	<10	20	160	survived
Mouse 8	<10	20	40	<10	20	40	survived
***MVA Vaccine***	***Challenge virus***						
**Wildtype**	CHIKV-S27			CHIKV-IND/NL10			
Mouse 1	<10	<10	<10	<10	<10	<10	dead
Mouse 2	<10	<10	<10	<10	<10	<10	dead
Mouse 3	<10	<10	<10	<10	<10	<10	dead
Mouse 4	<10	<10	<10	<10	<10	<10	dead
Mouse 5	<10	<10	<10	<10	<10	<10	dead
Mouse 6	<10	<10	<10	<10	<10	<10	dead
Mouse 7	<10	<10	<10	<10	<10	<10	dead
Mouse 8	<10	<10	<10	<10	<10	<10	dead

### MVA vaccines protect against lethal challenge with CHIKV-S27

**Figure 3:**
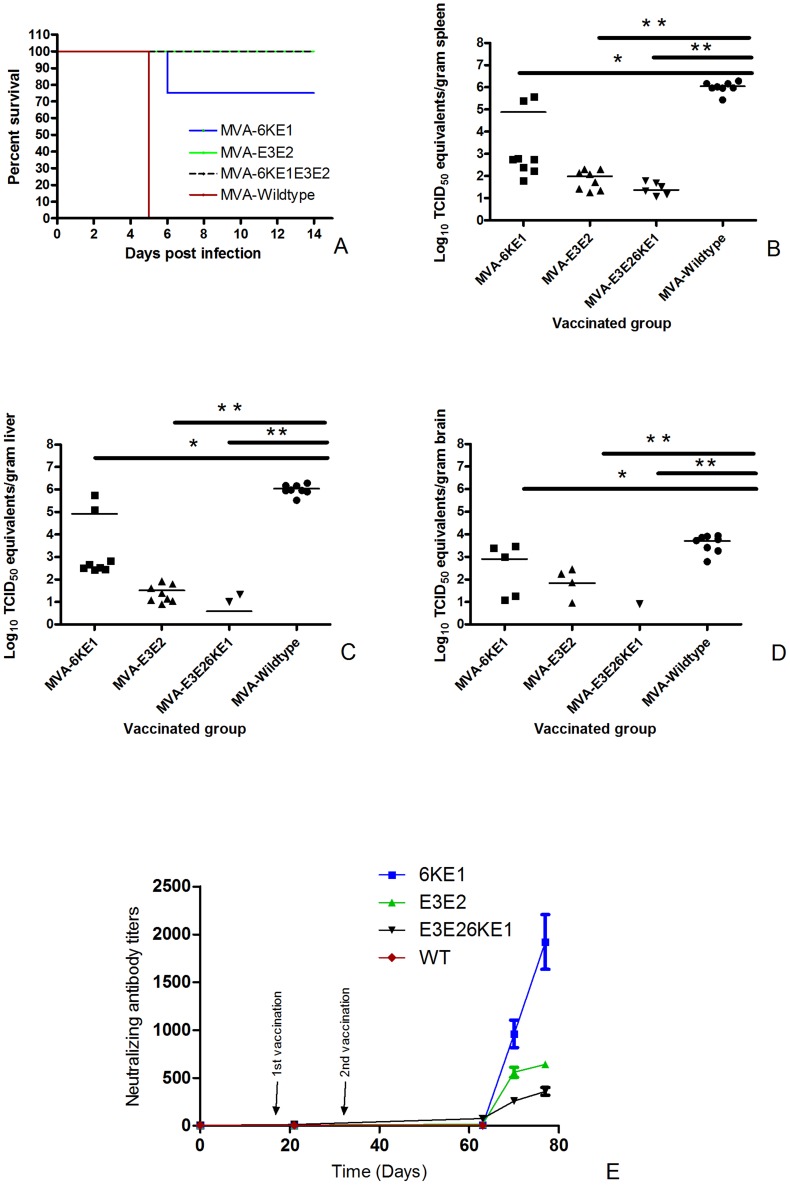
Survival of mice after vaccination and challenge infection with CHIKV. (**A**). Mice (n = 8) were immunized intra-muscularly with MVA-6KE1, MVA-E3E2, MVA-E3E26KE1 or MVA-wt. Subsequently, the mice were challenged intra-peritoneally with 1000 TCID_50_ CHIKV-S27. The survival rates of the mice after challenge are depicted as Kaplan-Meier curves. Differences between the curves were determined by the log-rank test. (**B, C, D**). Vial RNA copies were determined in spleen (B), liver (C) and brain (D) samples of animals that succumbed to the infection and survivors (day 14 post challenge). (**E**) Neutralizing antibody titers were determined on day 0, 21, 63, 70, and 77. Animals were immunized on day 0 and 21 and challenged on day 63. A clear booster response is seen after challenge, where a higher response was measured in MVA-6KE1 immunized group. The results are expressed as TCID_50_ equivalents per gram of tissue; *indicates a statistically significant result (*P*<0.05) and ** indicates highly significant results (*P*<0.001) as determined by the *Student's t test*.

In order to study the protective efficacy of the different recombinant MVA candidate vaccines, all the mice were challenged intra-peritoneally with a lethal dose of CHIKV-S27. The survival rates of the eight animals were monitored in each group after challenge. All mock-vaccinated (MVA-wt) mice died within five days post infection (Figure 3A). In the groups of mice vaccinated with MVA-E3E2 and MVA-E3E26KE1 respectively, all animals were protected against lethal infection. In contrast, 75% (6/8) of the animals immunized with 6KE1 were protected against lethal infection caused by challenge with CHIKV-S27. A clear booster response could be seen in all the groups (Figure 3E). The booster response was significantly higher in the group vaccinated with MVA-6KE1 Compared to the other groups. The anamnestic response in animals that received MVA-E3E26KE1 was two-fold lower than those immunized with MVA-E3E2, indicating that these two candidate vaccines provided similar levels of protection.

### MVA vaccines reduce viral load in organs of challenged animals

When animals reached the humane-endpoint after challenge, they were sacrificed and several tissues were collected to quantify virus titers. High levels of CHIKV RNA were detected in the liver and spleen (average: 10^5^ TCID_50_ equivalents/g tissue), and moderate amounts of viral RNA were found in the brain (average: 10^2^ TCID_50_ equivalents/g tissue) of mock vaccinated animals. However, no viral RNA was detected in the kidney or spinal cord of the infected animals (Figure 3 B–D). Six out of 8 animals immunized with MVA-E3E26KE1 and challenged with CHIK-S27 had very low levels viral RNA detectable in the spleen (range: 12 to 68 TCID_50_ equivalents/g tissue) and only 2 out of 8 animals had low RNA levels in the liver. No RNA was detected in the brain of these, indicating that MVA-E3E26KE1 provided high level protection against virus replication in the periphery and the brain. By contrast, most animals immunized with MVA-6KE1 and MVA-E3E2 and challenged with CHIKV-S27 had virus RNA in the liver, spleen and brain. The two animals that died after immunization with MVA-6KE1 had high levels of viral RNA in these organs (Figure 3). Infectious virus could not be recovered from any of the animals that received either MVA-E3E2 or MVA-E3E26KE1. On the other hand, low levels of infectious virus were isolated from the spleen of all the animals that received the MVA-6KE1 vaccine. However, the exact amount infectious virus in the spleen of these animals could not be determined because of toxicity in cell culture.

### Histological analysis

**Figure 4:**
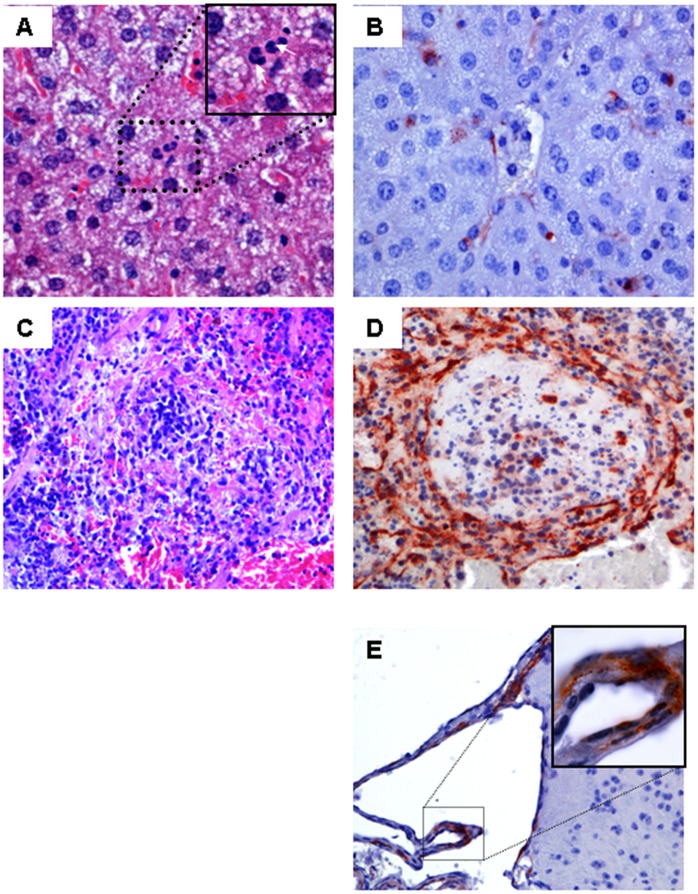
Histopathology of several tissues staining positive for CHIKV antigen. Panels A–E show representative staining of the liver, spleen, and brain of CHIKV infected AG129 mice. Hematoxylin and eosin-stained sections of the liver from all groups showed no abnormalities (A; 40× objective). Endothelial and Kupffer cells stained positive with anti-CHIKV capsid antibody (B, 40× objective). Massive depletion of lymphocytes was observed in the spleen of animals that died after challenge with CHIKV (C; HE staining, 40× objective). Antigen was mainly located in endothelial cells in the spleen (D). Epithelia cells of the choroid plexus (E) in the brain were scarcely stained and no antigen was found in the neuropil of the brain. Examples of positively stained cells are indicated by block arrows.

Immunohistochemical analyses were performed on tissues of animals that died as a result of CHIKV challenge and tissues collected from animals that survive the infection (day 14). No significant abnormalities were observed in the HE staining of the liver, brain, spinal cord, or leg skeletal muscle of neither the sick nor the healthy animals. In majority of the sick animals, neutrophils were seen in the sinusoids in the liver (Figure 4A). However, in the spleen extensive lymphocytic necrosis was observed in the white pulp of the control animals and in the MVA-6KE1 vaccinated animals that succumbed to infection (Figure 4C). This pathological finding was not observed in mice that survived the infection. Furthermore, abundant CHIKV antigens were detected in endothelial cells of all examined organs. In addition, antigen was detected in Kupffer cells in the liver (Figure 4B), macrophages and megakaryocytes in the spleen (Figure 4D) and adipocytes in the muscles of all mice that succumbed to infection. Few cells of the choroid plexus and endothelial cells were found antigen positive in the brain (Figure 4E), whereas no antigens were found in the spinal cord of any mice (not shown). Furthermore, no CHIKV antigen was detected in any organs of the animals that survived the infection. The negative immunohistochemical results obtained in the spleen of surviving animals that received MVA-6KE1, suggest low levels of virus in these animals.

## Discussion

In the present study, we have evaluated recombinant MVA expressing the structural genes of CHIKV-S27 for induction of protective immunity. Immunization of AG129 mice with MVAs expressing E2 or the entire CHIKV envelope polyprotein cassette E3E26KE1 provided 100% protection against lethal disease. Development of neutralizing antibodies correlated with protection against lethal infection *in vivo*.

To date, there are no licensed drugs or vaccines against CHIKV for use in humans. Safety and immunogenicity are major concerns when developing new vaccines. Recombinant vector vaccines such as MVA represent attractive alternatives for safe and effective vaccines. One theoretical disadvantage of an extensive use of the MVA system is the development of anti-MVA immunity. Several studies have shown that MVA is effective in eliciting protection against several virus infections and is not affected by anti-MVA immunity [41]–[43]. In this study, we have constructed several recombinant MVA vaccines that express E1, E2, or a combination of E1E3. We have chosen to clone the structural proteins from the West African CHIKV strain S27. E1 of S27 differs from la Reunion strain (LR2006) in only three amino acids (A226V, M270V, and V323A). These mutations are conserved. The E2 of S27 differed in six amino acids however (G57K, G79E, I211T, M266R, T312M, and A344T). The CHIK-NL10 strain was 100% identical to the LR2006 in the E1 protein and had only two mutations in the E2 (S191T and K252Q) compared to LR2006. However, because the S27 strain replicates faster in vitro and it is more virulent than NL10 in mice (not shown), we decided to use the S27 strain as the protein donor for the vaccine and challenge virus. A dose of 5×10^6^ TCID_50_ of recombinant MVA expressing E3E26KE1 rendered 100% protection against disease in challenged mice and the data suggest that presence of neutralizing antibodies correlates with protection. The antibody neutralization titers against a heterologous strain of CHIKV, which is more similar to LR2006, suggest that a vaccine based on the S27 strain would provide sufficient cross-protection against a different CHIKV strain. The relatively low levels of neutralizing antibody titers obtained against S27, NL10 and LS3 is unlikely to be explained by differences between the viruses. The most likely explanation is the relatively low dose of the vaccine that was administered, which was 1–2 log lower than what is normally given to reach high levels of antibody titers (10^8^ TCID_50_). The LS3 CHIKV strain is a synthetic virus based on the consensus sequence of several CHIKV strains. It is interesting to note that low levels of virus RNA could still be detected in 75% (6/8) of the spleens of animals immunized with E3E26KE1. Whether infectious virus persists is not known. It has been shown that CHIKV may persist in macrophages of humans and macaques long after acute infection [44]. Our results are in agreement with a recent study, which showed that MVA expressing C, E1, and E2 protected mice against lethal challenge with CHIKV [34]. Several other experimental vaccine candidates have been described for CHIKV, including inactivated [45]–[48], subunit protein [45], [49], virus-like particle (VLP) [50], [51], live-attenuated virus [37], [52], [53], DNA [54], [55] or vector vaccines [56]–[58]. All the platforms that express E1 and E2 rendered mice complete protection. It is known that vaccines based on inactivated virus or subunit proteins need to be adjuvanted to achieve satisfying immunogenicity. However, the use of adjuvants in humans is conflicting and non-adjuvanted vaccines are preferable. Live-attenuated vaccines have been shown to be safe and effective for several viral infections, but are associated with side-effects and there is fear for reversion to a virulent phenotype. Consistently, live-attenuated CHIKV vaccines have been shown to be more immunogenic in animal models and humans, but were associated with some side-effects [52]. DNA vaccines have so far not been particularly effective at generating antibody responses in humans [59], which is a concern as antibodies are believed to be required for protection against CHIKV infections [60], [61]. The role cell-mediated immunity cannot be excluded however. In this study we used AG129 mice, which are deficient in IFN-α, β, and γ receptor. It has been suggested that IFN-α and IFN-β system mainly inhibits early spread of virus from the primary site of infection, whereas the IFN-γ system may play a more important role in later stages of viral infection, e.g., viral persistence [36].

Our study further indicates that E2 is sufficient to induce complete protection in the mouse model. However, further studies are needed to see whether immunization with a single dose and whether use of a lower dose would provide clinical protection against homologous and heterologous strains of CHIKV.

## Supporting Information

Table S1 Sequence of primers and information about the amplified product.
(DOCX)
