# Supplementary material for: Recombinant Modified Vaccinia Virus Ankara Expressing Glycoprotein E2 of Chikungunya Virus Protects AG129 Mice against Lethal Challenge
Source: PLoS Negl Trop Dis. 2014 Sep 4;8(9):e3101. doi: 10.1371/journal.pntd.0003101 (PMC4154657; doi:10.1371/journal.pntd.0003101)
Supplement: Table S1 — Sequence of primers and information about the amplified product. (DOCX) [file pntd.0003101.s001.docx]

**Table S1.** Sequence of primers and information about the amplified product

| **set** | **target** | **Fw sequence** | **Rev sequence** | **fragment length** |
| --- | --- | --- | --- | --- |
| 1 | flank deletion site III | CACCAGCGTCTACATGACGAGCTTCCGAGTTCC | GAATGCACATACATAAGTACCGGCATCTCTAGCAGT | 750 bp + insert length |
| 2 | recombinant | AAAATGAAACCCGGGGATCT | GTCTGAGGAAAAGGTGTAGCGTAT | 552 bp (no insert) |
|  |  |  |  | 2034 bp (E3E2) |
|  |  |  |  | 3537 bp (E3E26KE1) |
|  |  |  |  | 2070 bp (6KE1) |
| 3 | mCHERRY | CGTGTGATATGAACAGACATTGTAT | AGGCTGAAGCTGAAGGACGG | 334 bp |
| 4 | E2 | CAGTCCGGCAACGTAAAGAT | CCCAAGCTT TTATTTAGCTGTTCTGATGCAGCA | 729 bp |
| 5 | E2-E1 | CCAAATTGTCCTGGTCTTCCT | CAGTCCGGCAACGTAAAGAT | 1505 bp |
| 6 | E1 | TCTTCAGCCTGGACACCT | TGCCTGCTGAACGACACG | 818 bp |
